# Combined Supplementation of Two Selenium Forms (Organic and Inorganic) and Iodine in Dairy Cows’ Diet to Obtain Enriched Milk, Cheese, and Yogurt

**DOI:** 10.3390/ani14091373

**Published:** 2024-05-02

**Authors:** Irene Azorín, Josefa Madrid, Silvia Martínez-Miró, Marina López, María Belén López, Miguel José López, Fuensanta Hernández

**Affiliations:** 1 Department of Animal Production, Faculty of Veterinary, Campus of International Excellence “Campus Mare Nostrum”, University of Murcia, 30100 Murcia, Spain; irene.azorin1@um.es (I.A.); silviamm@um.es (S.M.-M.); marina.lopez9@um.es (M.L.); mjlopeza@um.es (M.J.L.); nutri@um.es (F.H.); 2 Department of Food Technology, Nutrition and Bromatology, Faculty of Veterinary, Campus of International Excellence “Campus Mare Nostrum”, University of Murcia, 30100 Murcia, Spain; mbelen@um.es

**Keywords:** dairy product, feed additive, dairy cattle, micromineral

## Abstract

**Simple Summary:**

Selenium and iodine are two microelements necessary for a normal thyroid hormone metabolism, and their deficiency in human diets can result in a significant health problem. Milk is a good vehicle for mineral enrichment and an available food for the general population. Thus, the main objective of this study was to evaluate the combined dietary intake of iodine and two chemical forms of selenium in dairy cows, at the maximum levels permitted in the European Union, in order to obtain milk, cheese, and yogurt naturally enriched in these microminerals. As a result, animal performance and the overall health status of cows were not negatively affected by the diets. However, selenium and iodine were successfully transferred to milk, increasing the level of both minerals in cheese and increasing the iodine level in yogurt, without harming the quality or acceptance. Therefore, the value-added products obtained could contribute in a realistic way to improve the current supply in the market to enhance the intake of selenium and iodine in the human population.

**Abstract:**

This study evaluated the effects of dietary supplementation in dairy cows with two Se forms (organic and inorganic) and I at the maximum levels permitted in the European Union, with the aim to obtain naturally enriched milk and derived products. A total of 20 Holstein Friesian cows in lactation were fed 2 diets for 64 days: a control diet with a supply of 0.57 mg of inorganic Se and 0.57 mg of I per kg of ration in dry matter (DM), and an experimental diet (SeI) with a supply of 0.34 mg of inorganic Se, 0.23 mg of organic Se, and 5.68 mg of I per kg of ration in DM. The SeI diet did not modify the performance or, in general, the metabolic profile of cows. Se and I levels in milk were affected by diet type and time of measurement (*p* < 0.01). Thus, a marked increase of both microminerals was evident between the beginning and the end of the test, when the SeI diet was administered. For Se, this increase ranged from 1.95 to 3.29 μg/100 g of milk; and for I, from 19.69 to 110.06 μg/100 g of milk. The SeI diet increased (*p* < 0.01) the Se and I content in the cheese, reaching levels of 16.4 μg/100 g for Se and 269.7 μg/100 g for I. An increase in I was observed in yogurt from the SeI diet (*p* < 0.001). The supplementation of two forms of Se and I in the cows’ ration, at the levels evaluated, produced milk and dairy products enriched in these microelements without altering their quality parameters. However, a responsible intake of these products is necessary to avoid risks of deficiencies or excesses that could negatively affect the health of consumers.

## 1. Introduction

Selenium (Se) and iodine (I) are two of the essential microminerals with the greatest nutritional relevance in living organisms [1,2]. Both elements work together to provide normal thyroid hormone metabolism [3,4], making joint supplementation an interesting feeding option [5,6]. Iodine is an integral component of thyroid hormones [2] and selenoproteins protect the thyroid from the oxidative stress incurred during the synthesis process of these hormones [7]. At present, combatting Se and I deficiencies remains a challenge across the globe due to the low content of these elements in crops and forage from deficient soils worldwide [6]. Micronutrient deficiencies have a considerable negative impact on society, and the enrichment of food has become one of the most important and well-studied strategies to overcome this problem, which can also considerably affect farm animals, leading to significant economic losses [8,9]. In this context, Se and I are commonly supplemented in livestock feed to meet the dietary requirements of animals [5,10]; however, Se is regularly supplied in the form of inorganic salts, mainly sodium selenite, which is less bioavailable than organic sources, such as Se-enriched yeast [9,11,12]. However, excessive Se supplementation in the diet can cause toxicity, thus the signs of toxicity of Se generally begin to appear at Se levels of 5 to 8 mg/kg DM [1]. Also, I could cause toxicity. Negative effects start to be observed in beef cattle at levels > 50–100 mg/kg DM [13], although dairy cows seem more tolerant because part of the I excretion is carried out through milk [14].

Over the years, milk and its derived products have been an important focus of study as vehicles for mineral enrichment due to their availability and unique nutrient composition of high biological and nutritional quality [8,15,16]. The development of new functional foods is linked to healthier lifestyles, or specific nutritional needs [17]. These products’ differentiation has implications for the dairy production sector and its marketing, and could even lead to a potential economic benefit [17,18,19]. Milk is a daily consumed food among the general population and it contains a high nutritional density; it is the main dietary source of important elements such as calcium or phosphorus [20]. However, dairy foods represent deficient sources of Se, and I is naturally present at low levels in milk, despite it being one of the major dietary sources of I [8,21]. Nevertheless, both I and organic sources of Se can be transferred very efficiently to milk through dairy cows’ diets due to the rapid incorporation of Se into milk proteins [22] and the ability of I to accumulate in the mammary gland [2]. Therefore, these sources of food are interesting to study in terms of their enrichment using these minerals in particular. Despite all the reported benefits of organic Se supplementation in dairy cows’ diets, numerous studies appear to remain inconclusive due to the influence of several physiological and productive factors on this outcome [23]. In addition, further research is necessary to investigate the interaction between organic sources of Se and I when the two elements are simultaneously supplemented in ruminant feeds, as well as their transfer to milk and milk products. From this perspective, authors, such as Moschini et al. [4], studied the combined supplementation of these elements in cow rations and the carry-over effects on milk and cheese products, although they only used inorganic sources. Since the European Union has limited the source types and the concentrations of Se and I in animal diets, the study of the combined use of these elements in dairy cow rations at the highest levels permitted (adding part of the Se in an organic form) would not only determine the effect of this strategy on animal production and health but also the capacity to produce dairy products enriched in both microelements under European animal production laws. Also, it should be noted that there are indications of the “Upper Intake Level” (UL) for Se and I in humans to avoid excesses. In this regard, for Se, it is set at 255 μg/day [24], and for I at 600 μg/day [25].

Thus, the hypothesis of this work suggests that, after supplementing the cows’ diet with organic and inorganic Se and high doses of I (around the maximum level permitted by the European Union legislation), higher levels of these minerals are expected to be present in both the cows’ milk and derived products, possibly achieving a labelable status that would result in the popularity of these naturally enriched products of animal origin in the current market. Therefore, this study focuses on assessing the combined supplementation of 0.5 mg of Se (0.3 plus 0.2 mg of inorganic and organic Se, respectively) plus 5 mg of I per kg of ration (values expressed for a complete diet with a moisture content of 12%, according to the legislation) in the diet of dairy cows in order to evaluate its effects on production parameters, blood and milk profiles, as well as the composition and quality of dairy products.

## 2. Materials and Methods

Animal handling and experimental protocols were performed in accordance with the Code of Good Research Practices of the University of Murcia (Murcia, Spain), with a protocol number of A13170805, under the European Union regulation (“European Union Directive 2010/63/EU”) in order to guarantee the best scientific practices based on the appropriate ethical and legal standards [26].

### 2.1. Design of the Trial

A total of 20 multiparous Holstein Friesian cows in the lactation stage with an average live weight of 650 kg, 170 days in milk, and a 33 kg/day milk yield were allocated to 2 random groups composed of 10 cows. A total of 2 separate areas were prepared for the animals, where they were provided with straw litter, troughs with drinking water, and feeders (30 cm/cow) for their rations. The trial was performed over a period of 64 days at the facilities of the Veterinary Farm of the University of Murcia.

Two micromineral feeding treatments were provided to each group of cows: a control treatment (CON) with a supply of 0.57 mg of inorganic Se plus 0.57 mg of I/kg of DM ration, and an experimental treatment (SeI) with a supply of 0.34 mg of inorganic Se plus 0.23 mg of organic Se and 5.68 mg of I/kg of DM ration. Furthermore, the European Union legislation states that 0.5 mg of Se (0.3 mg plus 0.2 mg of inorganic and organic Se, respectively) plus 5 mg of I per kg of ration (values expressed as the complete diet with a moisture content of 12%) are the maximum levels permitted for use [27,28]. Both treatments offered a similar totally mixed ration (TMR), but with a different vitamin–mineral premix that included Se and I supplements for the respective rations (Table 1).

The source of inorganic Se was sodium selenite, and the organic Se source was Se yeast Sel-Plex^®^ (Alltech, Nicholasville, KY, USA), which was rich in organic Se obtained from a particular *Saccharomyces cerevisiae* strain (CNCM I-3060) that contained more than 63% of selenomethionine (SeMet). In addition, potassium iodide (3b201) was used as the I source. According to the nutritional requirements for mid-lactation cows described by the Fundación Española para el Desarrollo de la Nutrición Animal (FEDNA) [30], both diets were designed to provide equal levels of energy and nitrogen (6.56 MJ ENl/kg of DM and 156 g CP/kg of DM). A total of 22.5 kg of TMR (DM) was offered to each animal twice a day after each milking. Table 2 presents the chemical composition of the experimental diets.

The cows were milked twice every day at 07:00 h and 19:00 h. An iodine-free solution was used as a post-milking treatment. On days 0 (T_0_) and 64 (T_64_), the individual milk production value was registered, and individual samples were collected using the hand milking method. A total of 150 mL was collected for pH determination; analyses of protein, fat, and lactose; and somatic cell count (SCC) purposes. Additionally, 100 mL of milk was stored at −20 °C for the subsequent determination of the mineral content (Se, I, Ca, P, Zn, and Cu). For the manufacture of dairy products (spray-dried milk, cheese, and yogurt), 20 L of milk from both treatments was collected prior to the end of the test, on Days 50, 57, and 64.

Samples of blood were collected from all cows using coccygeal venipuncture, on Days 0 and 64, using VACUETTE^®^ tubes with lithium heparin (Greiner Bio-One GmbH, Kremsmünster, Austria) prior to offering the animals the first TMR of the day. The whole blood was used to analyze the mineral content (Se, Ca, P, Zn, and Cu) and GSH-Px activity, whereas the metabolic profile and thyroxine hormone (T_4_) determination were analyzed in plasma samples, which were frozen at −80 °C until they were processed.

The body condition of cows was recorded on Days 0 and 64, in a range of 1 (severe underconditioning) to 5 (severe overconditioning), following the descriptions provided by Edmonson et al. [31].

### 2.2. Spray-Drying Process

The spray-drying operation was performed on fresh milk samples using a Büchi B290 mini-spray dryer (Büchi, Flawil, Switzerland) by applying the following conditions: an inflow temperature of 220 °C, flow rate of 439 L/h, maximum step volume of 32 m^3^/h, sample input flow of 3 mL/min, and outlet temperature of 154 °C. A total of 3 L of raw milk was atomized per treatment and batch, and these samples were then chemically analyzed.

### 2.3. Cheese and Yogurt Manufacture

For fresh cheese manufacture, the milk obtained was pasteurized at 75 °C for 20 s in an experimental semi-industrial scale plate heat exchanger with a 5 L capacity (Redefine Food Solutions, S.L., Bullas, Murcia, Spain). After the pasteurization process, 10 L of milk was placed in a 12 L double-circle tank (“double 0”) stainless-steel vat (Pierre Guerin Technologies, Mauzé, France). Anhydrous CaCl_2_ (Chr. Hansen, France) and calf rennet with 80% chymosin and 145 IMCU/mL (Caglio Star España, S.A., Murcia, Spain) were added with agitation in a proportion of 0.3 mL/kg after tempering the milk until it reached a temperature in the range of 33–34 °C. After the product was rested for 40 min, it was cut for 3 min at a cutting speed of 25% and was left to stand for 5 min. Thereafter, 80 g of salt was added, and the solution was stirred again for 1 min. Subsequently, the product was left to rest for another 10 min and then stirred again for 1 min. The whey was drained and the curds were filled into molds without being pressed. Finally, the cheese samples were left to drain in a refrigerator at a temperature of 4 °C for a period of 24 h. Four cheeses per treatment and batch were produced, using half of each cheese to perform a physicochemical analysis and the other half for a sensory study.

For the yogurt-manufacturing process, the milk was pasteurized at 95 °C for 240 s using the same plate heat exchanger. After the pasteurization stage, 1 L of pasteurized milk was placed in a stainless-steel tank with the addition of 20 g of sugar and 100 g of milk powder, which were then mixed and heated to 95 °C. This was followed by the product being tempered to reach a temperature of 45 °C, and the additions of *Streptococcus thermophilus* and *Lactobacillus bulgaricus* ferment (YOMIX-300, Chr-Hansen, Madrid, Spain) in a proportion of 1 g/100 L. The final step was the packaging of the yogurts, which were kept at 45 °C for 4 h and then stored at 4 °C after the incubation phase. Six yogurts were prepared per treatment and batch, using half of the yogurts for the physicochemical analysis and the other half for the sensory study.

### 2.4. Laboratory Procedures

#### 2.4.1. Diets Analysis

The TMR diets were sampled and analyzed every week. Pre-drying was performed in a convection oven at 60 °C for 48 h, and the samples were subsequently ground through a 1 mm sieve (Retsch ZM 200 Ultra Centrifugal Mill; Retsch, Hann, Germany). Diet samples were analyzed according to the procedures of the Association of Official Analytical Chemists (AOAC) [32]: ash content (942.05 method), ether extract (EE) (920.39 method), and crude protein (CP) (2001.11 method). The methods of Van Soest et al. [33] were used to measure the neutral detergent fiber (NDF) and acid detergent fiber (ADF) contents. Acid detergent lignin (ADL) was determined by solubilizing cellulose with 72% sulfuric acid.

#### 2.4.2. Mineral Assay (Se, Ca, P, Zn, Cu, and I)

The whole blood, diet (TMR), fresh milk, spray-dried milk, and cheese and yogurt samples were hydrolyzed with HNO_3_ in a microwave digestion system (Milestone Ethos X Microwave, Sorisole, Italy). Se, Ca, P, Zn, and Cu were quantified for the samples (except for TMR, where Zn and Cu were not evaluated) using inductively coupled plasma–mass spectrometry (Agilent 7900 ICP-MS, Santa Clara, CA, USA) by applying the standard addition method. For the determination of I in the TMR, fresh milk, spray-dried milk, and cheese and yogurt samples, alkaline hydrolysis with an aqueous ammonia solution was performed prior to the microwave digestion step, following a method adapted from Meyer et al. [34].

#### 2.4.3. GSH-Px and Metabolic Profiles

In the whole blood samples, glutathione peroxidase (GSH-Px) was determined with a commercial kit (Ransel test kit, Randox Laboratories Ltd., Crumlin, UK) adapted from the method of Paglia and Valentine [35]. For the determination of hemoglobin (Hb), a hematological analyzer (Advia 120, Siemens Healthcare Diagnostics SL, Barcelona, Spain) was required. The assessment of the overall metabolic profile in the plasma samples was performed using an automatic chemistry analyzer (Olympus AU600, Olympus Diagnostica Europe GmbH, Ennis, Ireland), and commercial Beckman kits (Beckman Coulter Inc., Fullerton, CA, USA) were used for the assays. Glucose was measured using the hexokinase G-6-PDH method; total triglycerides (TGs) were hydrolyzed using a combination of microbial lipases to produce glycerol and fatty acids; urea was measured using the enzymatic adapted Talke and Schubert [36] method; total proteins were measured using the adapted Weichselbaum [37] method; and cholesterol was determined using the cholesterol dehydrogenase method. TAC (antioxidant capacity) and TOS (oxidative status) were analyzed in the plasma samples using the methods described by Erel et al. [38,39]. T_4_ total hormone was determined with the immunoassay of chemiluminescence (Immulite 1000 Immunoassay System, Siemens Medical Solutions Diagnostics, Deerfield, IL, USA).

#### 2.4.4. Composition and pH Outcomes of Fresh Milk, Yogurt, and Cheese

The compositions of fresh milk (fat, protein, lactose, and non-fat solids) and yogurt (fat and protein) were analyzed with infrared spectroscopy (MilkoScan FT6000, Foss Electric, Hillerod, Denmark) following the International Dairy Federation (IDF) Standard (141B:1996) [40]. In the fresh milk, the SCC was determined using fluorescence and flow cytometry based on the manufacturer’s FOSS method (Fossomatic TM 5000, Foss Electric, Hillerod, Denmark). For the cheeses, the dry matter was determined in the grated samples (3 g ± 10 mg), which were dried to achieve a constant weight according to IDF Standard 4:2004 [41]; the fat content was calculated using the IDF Standard 5:2004 procedure [42]; and the protein content was determined using the Kjeldahl method (IDF Standard 25:2008) [43].

For the pH determination of both the milk and yogurt samples, a Crison^®^ pH meter (micro–pH 2001, Barcelona, Spain) connected to a Crison^®^ glass combination electrode (1952–2002) previously calibrated at room temperature was used. For the pH determination of cheese, grated samples (5 g ± 0.1 mg) were suspended in 30 mL of distilled water and shaken for 10 min before performing the measurement.

#### 2.4.5. Physical Parameters of Yogurt and Cheese

Yogurt syneresis was obtained by measuring the whey present in 50 g of a sample centrifuged at 3000 rpm for 20 min. The cheese yield was determined as the kilograms of cheese obtained from 100 kg of milk. The water activity of the cheese was determined using Novasina Thermoconstanter equipment (TH-2, RTD-200 model, Novasina AG, Lachen, Suiza), and measurements were performed in triplicate at a room temperature of 25 °C. The color/lightness attributes were determined for the cheese and yogurt with a CR-400 MINOLTA colorimeter (Konica Minolta Sensing, Ramsey, NJ, USA). Three measurements of luminance (L*), redness (a*), and yellowness (b*) were obtained per replication.

The cheeses were tested in a texture analyzer (TA-XT Plus, Stable Micro Systems Ltd., Godalming, UK) fitted with a 500 N load cell to obtain the results of the texture parameters. Analyses were performed on 3 cm^3^ cube-shaped cheese samples, without the rind, at an ambient temperature of 20 °C. For the TPA tests, each sample was compressed twice to a size of 15 mm using a P/100 probe (compression platen with a 100 mm diameter) moving at a speed of 1 mm/s. The results were processed with the software Exponent (version 5.1.1.0). The texture parameters we determined included the hardness (expressed as N), cohesiveness (dimensionless), gumminess (product of hardness and cohesiveness, expressed as N), elasticity (expressed as mm), chewiness (product of gumminess × elasticity, expressed as N mm), and adhesiveness (expressed as N s), calculated as described by Bourne [44].

#### 2.4.6. Sensorial Parameters of Dairy Products

Ten trained panelists conducted a sensory analysis of the cheese and yogurt samples, which was repeated in three separate sessions. In cheese, the properties tested were the odor, cream odor, cow milk odor, cow milk flavor, cream flavor, salty flavor, other flavors, consistency, graininess, juiciness, fatty nature, and overall acceptability; for yogurts, the attributes tested were the consistency, odor, acid odor, cow milk odor, acid flavor, cow milk flavor, cremosity, metallic flavor, astringency, other flavors, and overall acceptability. Half of each cheese was divided into approximately 1-centimeter-thick portions, and the yogurts were served in individual plastic cups marked with random digits. A scoring system with a structured intensity scale (1–9 for cheese and 0–10 for yogurt) was used to conduct the sensory analysis. Salt-free crackers and mineral water were served to remove any aftertaste between taste-testing the samples.

### 2.5. Statistical Analyses

IBM SPSS Statistics software (IBM Corporation, Armonk, NY, USA) was used to perform the statistical analyses. The relevant data were assessed for normality using the Shapiro–Wilk test and those that presented non-normal distributions were log-transformed. Whole blood, plasma, and milk were then analyzed using a general linear model with repeated measures, where the type of treatment (CON or SeI) was considered as an inter-subject factor, and the time of measure performed on different trial days (T_0_ and T_64_) was considered as an intra-subject factor. The physicochemical and sensorial data for the cheese and yogurt were analyzed using the mixed procedure of the same statistics software, considering the type of treatment as a fixed effect and the elaboration batch as a random effect. The results were considered statistically significant at *p* < 0.05.

## 3. Results

### 3.1. Production Parameters and Mineral and Metabolic Profiles of the Blood

Se and I supplementations did not affect the body condition score or daily milk production of the dairy cows under study (Table 3). Neither interaction was observed between the type of diet and the time for these parameters (*p* > 0.05).

The dietary treatments did not affect (*p* > 0.05) the contents of Ca, P, Cu, Zn, and Se in the whole blood samples of the lactating cows (Table 4). At the end of the test (Day 64), higher levels of Ca, Cu, Zn, and Se were observed in the cow whole blood samples (*p* < 0.05).

In our study, the type of ration did not affect the GSH-Px levels in the blood samples. However, a significant day effect was observed, with higher levels of GSH-Px activity being observed at the end of the experimental period compared to the beginning of it. In addition, when we expressed the GSH-Px blood content as g Hb, a significant interaction was observed between the type of diet and the day, indicating that this activity was greater at the end of the test in the SeI cows.

In the plasma samples, regarding the levels of glucose, TGs, urea, total protein, and cholesterol, differences were only registered for the glucose, with the day effect (*p* < 0.05) being lower in both groups at the end of the study, and the same was observed for the total protein content (*p* < 0.01). For the glucose, this effect was more pronounced in the control diet (interaction trend, *p* = 0.078), and the trend of a higher glucose level was also observed for the SeI-diet group (*p* = 0.066).

An increasing trend for total antioxidant status (TOS) at the end of the experimental period (*p* = 0.069) was observed. In addition, an interaction between the dietary treatment and time was observed for the plasma T_4_ hormone (*p* < 0.05) because the T_4_ hormone decreased at the end of the study but only in CON cows.

### 3.2. Fresh Milk Analyses

Table 5 provides the results for the SCC and chemical and mineral compositions of the milk collected from cows subjected to both treatments. In our study, we observed that the SCC was higher in both groups at the end of the experimental period (*p* = 0.05), and we did not observe the occurrence of dietary treatment effect or interaction between the sampling day and the type of ration.

Significant differences were observed regarding the chemical composition of the milk at the end of the experimental period, where it increased in ether extract (*p* < 0.05), crude protein (*p* < 0.001), non-fat solids (*p* < 0.01), and decreased in lactose (*p* < 0.001); however, these parameters were not influenced by the type of ration or the existence of interactions. For the pH of the milk, no significant effect of the factors studied was observed; it only presented a slight tendency (*p* = 0.076) to decrease at the end of the examination period.

When we assessed the mineral profile of the milk, we observed that the Ca and Cu contents were not affected by the factors studied, but the Zn level increased significantly (*p* < 0.01) at the end of the test, showing a slight tendency (*p* = 0.087) toward presenting a higher value for the SeI-diet cows. Phosphorus behaved similarly to Zn, although an interaction between the dietary treatment and time (*p* < 0.05) was observed, such that the increase in the P content at the end of the experiment was more evident in the SeI treatment.

In relation to the contents of Se and I, it was observed that the supplementation of the ration with organic Se and high doses of I presented a significant increase in these elements in milk. In addition, an effect of the sampling day (*p* < 0.01) was also observed, as well as the presence of an interaction between the dietary treatment and time (*p* < 0.01) for both minerals.

Thus, a very marked increase in both minerals was evidenced at the end of the study when the diet supplemented with organic Se and high levels of I was administered to the cows; for Se, this increase occurred from 1.95 μg/100 g on Day 0 to 3.29 μg/100 g of milk on Day 64, and for I, a level more than 5 times higher was achieved (19.69 versus 110.06 μg/100 g of milk for Days 0 and 64, respectively).

### 3.3. Spray-Dried Milk Analyses

Table 6 presents the mineral composition of the milk after it was subjected to the spray-drying process. No effect of the dietary treatment of the animals was observed on the Ca, P, Cu, and Zn levels in the processed milk; however, we determined that the Se and I were affected by the type of ration that the animals consumed. Thus, the Se content of spray-dried milk increased three 3 when the cows were fed with the SeI ration compared to the control (22.66 versus 6.92 µg/100 g of spray-dried milk, respectively). For the case of I, we observed that this increase was 6 times higher for the diet supplemented with organic Se and high doses of I compared to the other group (1568.65 versus 245.47 µg/100 g of spray-dried milk, respectively).

### 3.4. Cheese Analyses

Table 7 presents the chemical and mineral compositions of the fresh cheese obtained from the experimental treatments. Dietary supplementation with organic Se and I increased the dry extract (*p* = 0.061) and Se and I content of fresh cheese (*p* < 0.01). The ration had no effect on the protein, Ca, P, Cu, and Zn contents of the fresh cheese.

The dietary treatment did not affect the pH value, milk yield, water activity, or colorimetric parameters (*p* > 0.05). In addition, in the texture profile, the adhesiveness parameter (*p* = 0.078) tended to decrease in the SeI fresh cheese group.

**Figure 1:**
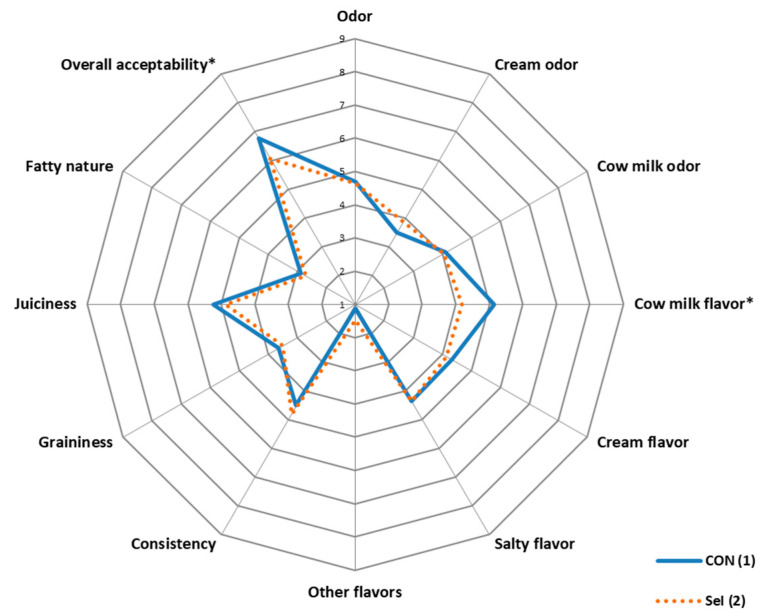
Sensory profile (1–9 scale) of fresh cheese obtained from cows fed with the experimental treatments. Spider chart: (1) CON Ration. (2) SeI Ration. The asterisk (*) indicates that there are differences (*p* < 0.05) between treatments.

The results of the sensory profiles of the fresh cheeses are exhibited in Figure 1. Fresh cheeses produced with milk obtained from the batch supplemented with organic Se and I (SeI) had a milder cow milk flavor (*p* < 0.05) and lower overall acceptability value (*p* < 0.05).

### 3.5. Yogurt Analyses

The diet supplementation performed with organic Se and I did not affect the pH; crude protein; ether extract; syneresis; or Ca, P, Cu, Zn, and Se contents of the yogurts (Table 8). However, the I yogurt content increased (*p* < 0.001), being approximately 4 times higher in the SeI group.

**Figure 2:**
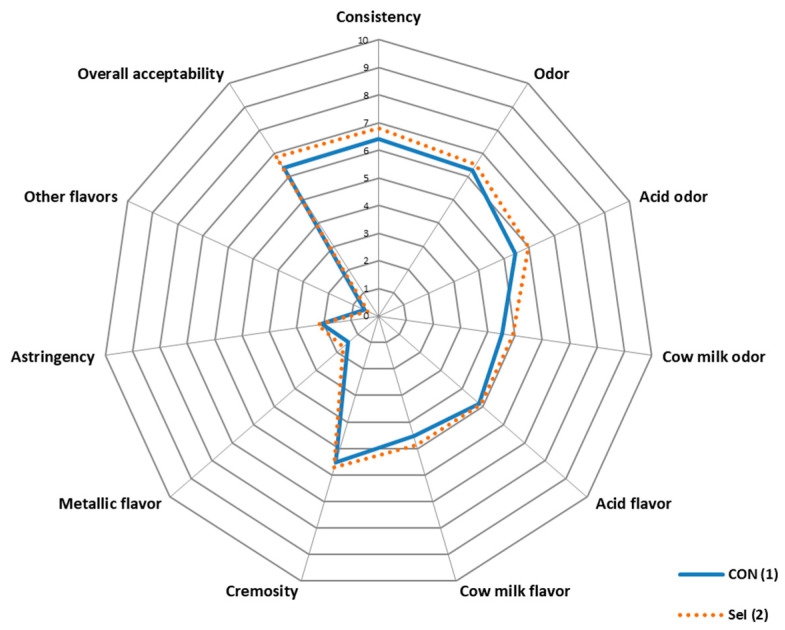
Sensory profile (scale 0–10) of yogurt obtained from cows fed the experimental treatments. Spider chart: (1) CON Ration. (2) SeI Ration.

Figure 2 shows the results of the sensory study of the yogurts, where no differences were observed between treatments.

## 4. Discussion

This study focused on the evaluation of combined Se (organic and inorganic) and I supplementation in dairy cow diets, using the maximum levels allowed by European Union legislation, in order to assess its effects on the productive parameters and the mineral and metabolic status of the animals, as well as on the composition and quality of milk, cheese, and yogurt. In addition, this study explores the potential for specific labeling of dairy products derived from this feeding strategy.

### 4.1. Chemical Composition of Experimental Diets

Despite the complexity of TMR sampling, the analyzed values for crude protein and neutral detergent fiber and other nutrients were very similar to those expected in the formulated diets. Regarding the analyzed micronutrients, the control and SeI diet reached very similar Se values (0.56 and 0.58 mg/kg DM, respectively) to those expected (0.57 mg/kg DM). However, the I value analyzed in the rations (0.95 and 6.58 mg/kg DM, respectively) were slightly higher than expected (0.57 and 5.68 mg/kg DM, respectively) but an approximate increase of 5 mg/kg was maintained between the 2 diets.

### 4.2. Production Parameters and Metabolic Status

In the field of dairy farming, the study of production parameters is important, since a reduction in milk production is one of the most economically critical issues associated with Se deficiency, presenting significant impacts on farms [45]. From our experience, we observed that a daily intake of 11.5 mg of Se and 112.5 mg of I did not influence the milk yield and body condition of cows. From this perspective, Givens et al. [46] also observed no differences in the cows’ body condition following Se intake but noted that increasing the dietary Se level from 9.4 mg/day to 14.3 mg/day, regardless of the Se source, resulted in a 2 kg/day decrease in milk production. However, in our test, the daily Se consumption was lower and similar between diets where we only changed the Se source. In contrast, in a study conducted by Bagnicka et al. [47], the authors observed that the milk volume produced after 90 days of supplementation with Se yeast, with an ingestion amount of 6 mg Se/day, was approximately 20% higher than in the group supplemented with an inorganic Se source. On the other hand, in another study, Iannaccone et al. [48] observed no variations in the milk yield with a daily I intake of 85 mg/day, and Kaufmann and Rambeck [49] did not observe any differences in body weight gain and feed intake after supplementing the cows’ diets with 150 mg I/day. As for the joint supplementation, Moschini [4] did not determine any effects between the milk yield and the levels of Se and I added to the feed, with maximum intake levels of 11.30 mg Se/cow/day and 82.17 mg I/cow/day being obtained. This result is in line with our results, even though these authors only used inorganic sources of food.

Moreover, we determined that diet supplementation with organic Se and I did not affect the levels of Ca, P, Cu, Zn, and Se in the sampled blood. However, the day effect on Ca, Cu, Zn, and Se levels recorded at the end of this study can probably be associated with factors related to the stage of lactation or production, and these changes in blood trace element concentrations are not uniform among herds [50,51]. Several regulation points exist in the transfer of minerals to the blood, including intestinal absorption, which plays an important role in regulating the homeostatic control of Zn and Cu. Moreover, during the milk synthesis stage, there is an increase in the demand for Ca, in addition to a relatively slow response to the regulation of Ca absorption from the intestinal tract [51].

On the other hand, the level of Se in blood depends mainly on recent dietary intake and the bioavailability of the form of supplemented Se [52] since transformation pathways that take place in the rumen are highly dependent on the type of Se chemical compounds [53]. Inorganic salts, such as sodium selenite, are highly susceptible to dissolution and form elemental Se in the rumen environment [54], are poorly absorbed with a short half-life in the body, and are mostly excreted in feces [12,55] while organic sources such as Se yeast improve the bioavailability of Se, increasing its incorporation into the microbial protein as seleno-aminoacids in rumen metabolism [54,56]. Several studies observed a higher Se content in the blood samples tested when Se yeast, instead of sodium selenite, was added to the cows’ diets [47,54,57,58] since organic Se forms more efficiently accumulate in tissues, being more bioavailable and less toxic than inorganic forms [12]. However, we observed that, although one of the diets was supplemented with an organic source of Se, we did not view significant differences in the blood Se levels. The same result was reported by Azorín et al. [59] after supplementing cows’ diets with moderate levels of organic and inorganic Se. This result can be explained by the fact that the incorporation of Se into erythrocytes occurs during cell synthesis, acting as a long-term marker of the nutritional status of this mineral and reflecting a more chronic Se status [54,60]. In addition, the lack of a response can be associated with the fact that the total dietary Se intake was above the threshold at which a response would be recorded, as was evident in the study conducted by Juniper et al. [61].

Selenium contributes substantially to the antioxidant defense network [62] and forms part of the active center of many antioxidant enzymes, such as glutathione peroxidase (GSH-Px) [22], whose activity depends on the level of Se in feed and is considered a reliable indicator of Se uptake and its concentration in whole blood [61,63]. In our study, a day effect was observed at the end of the test in relation to the GSH-Px levels, and an interaction between the ration type and time was also observed when GSH-Px was measured in relation to hemoglobin, this effect being greater in the group supplemented with organic Se and I. Sun et al. [58] employed a similar approach and observed an improvement in the antioxidant capacity of dairy cows with an increase in serum GSH-Px after Se yeast supplementation. In contrast, Juniper et al. [61] reported no significant treatment effects on the GSH-Px level, and they hypothesized that this could have been due to the brief trial period that did not provide enough time for any differences in the GSH-Px activity to develop. In addition, in our study, we did not observe any differences in the total antioxidant capacity (TAC) or total oxidant status (TOS), but an increasing trend for the TOS at the end of the experimental period was observed.

Similar to the mineral content in blood, the differences we observed in some plasma metabolites at the end of the test could be mainly related to the lactation stage, where serum protein, albumin, and glucose could be subject to changes [64]. Only significant differences were registered for the glucose and total protein levels, which were lower in both groups at the end of the study but presented an increasing trend for the glucose level in the serum for the SeI-diet group. This trend to increase the glucose levels after Se supplementation is in accordance with Pamungkas et al. [65], who obtained higher glucose levels after supplementing animals’ diets with a mixture of Se and vitamin E. This result can also be related to the high-dose I supplementation scenario since Hillman and Curtis [66] observed that serum glucose increased after feeding cows with an average iodide amount of 164 mg/cow/day.

In ruminants, it has been determined that >70% of ingested I is absorbed in the rumen and omasum. However, in the abomasum, I secretion exceeds absorption, although significant I reabsorption occurs in the small and large intestines [67]. The tissue that accumulates the most I in cattle is the thyroid gland [34], where it is incorporated into the synthesis of thyroid hormones [68]. In addition, the mammary gland concentrates I, excreting it through milk. There is a strong correlation between the amount of I in the diet and the concentration of I in milk [69], and several studies indicate that total I excretion through milk ranges from 10 to 55%, depending on the level of I and the presence of anti-nutritional factors [68].

Selenium and I are both essential for normal thyroid hormone metabolism [3,4], with I acting as an essential substrate for thyroid hormone synthesis while the selenoproteins protect the thyroid from oxidative stress incurred during this process and regulate the number of active hormones [7]. An iodine–selenium imbalance affects the regulation of the hypothalamus-pituitary-thyroid axis and leads to diseases associated with multiple metabolic disorders [70,71]. To evaluate the effects of supplementation with organic Se and a high I level on the thyroid metabolism of cows, the level of total thyroxine (T_4_) hormone in cow plasma was determined. Se enzymes are responsible for the conversion of T_4_ in triiodothyronine (T_3_), the active metabolite that has a much shorter half-life than T_4_ [3,6]. Specifically, the enzyme responsible for this activation is 5-iodothyronine deiodinase, a Se-dependent protein [72]. Despite this behavior, long-term variations in dietary Se levels, such as a sustained deficiency over time, are necessary to observe the effects on thyroid hormone concentrations [73]. On the other hand, plasma T_4_ values tend to be relatively stable and hardly respond to short-term dietary I supplementation in cattle [74]. In our test, at the end of the experimental period, only an interaction between diet type and time was observed, with a decrease in the T_4_ hormone in the control group fed with low levels of I. Some researchers observed lower levels of the T_4_ hormone in goats prior to I supplementation, with trial periods over 50 days being conducted [75,76]. Moreover, it can be determined that the metabolism of thyroid hormones is very complex and multifactorial, making simple interpretations difficult to perform [76].

Currently, in animal production, both the health status and welfare of animals are of particular importance [77,78], not only from the point of view of the researcher, farmer, or livestock technician but also for their transfer of knowledge to society at large [79]. It should be noted that no pathology was observed during the trial and no negative effects on the physiological state of the animals were evidenced from the results obtained.

### 4.3. Milk

Despite the fact that many researchers have observed a reduction in the SCC in milk after Se and I supplementations [48,58,80], no influence of diet was observed in our study, and we perceived an increase in these levels at the end of the test, which could be associated with the lactation stage [81,82].

Milk composition varies substantially throughout the lactation period, mainly as a result of physiological changes occurring in a cow [81,83]. In our study, the differences observed in the chemical composition of milk at the end of the experimental period (increases in fat and protein and a decrease in lactose) were consistent with the results reported by other authors throughout the lactation period [84,85,86]. From this perspective, in a study conducted by Sun et al. [58], the researchers observed the day effect on protein, fat, lactose, and non-fat solids after supplementing cows’ diet with Se yeast at 0.5 or 5 mg/kg of DM, but with no treatment effect. Givens et al. [46] also did not observe any effects on the main chemical components of milk after supplementing cows’ diets with different Se concentrations (ranging from 0.38 to 1.14 mg/kg of DM). In contrast, Nudda et al. [87] observed a reduction in milk lactose levels in small ruminants when supplemented with 0.90 mg/day of potassium iodide compared with the control group supplemented with 0 mg/day and another group supplemented with 0.45 mg/day. Furthermore, they observed no effects on the protein level, but they did observe a reduction in the fat content after supplementation with 0.90 mg/day.

Milk mineral levels also varied throughout the lactation period [81], and this could explain the day effects observed for P and Zn, which resulted in an increase in the content of these minerals at the end of the test. Furthermore, the slight increase observed in the Zn level is in accordance with the result obtained by Bagnicka et al. [47], who observed higher levels of Zn after 90 days of supplementation with Se yeast. Some studies have shown that Se and I supplementation in feed affects the concentration of these trace elements in milk [4,12,22,47,68,80]. In a similar manner, Mehdi and Dufrasne [72] perceived that analyzing the Se content in cows’ milk is a simple way to assess the Se status of a herd, and Berg et al. [88] believed that the amount of I in cows’ milk reflected the dietary I content and was also an indicator of the I status of the animals.

Fresh milk obtained from the supplemented diet group had 208% more Se and 424% more I than the control group at the end of the test. This observation is in agreement with the result obtained by Barbé et al. [12], who determined the highest quantity of Se present in cow milk when they supplemented the diet with Se yeast, which, when compared with other Se sources, was the most efficient source to transfer Se to the milk. Similar values of milk Se concentration were reported by Givens et al. [46], who obtained a level of 28 μg/L after feeding cows with Se yeast in the amount of 0.4 mg/kg of DM. Bagnicka et al. [47] determined an increase of over 300% of Se in cow milk subjected to organic Se treatment compared to the inorganic source. This occurs because the mammary gland is one of the most active protein-synthesizing organs in cows, and the selenomethionine present in organic Se sources is largely incorporated into milk proteins in a nonspecific way, instead of methionine [12,22,89]. It is important to highlight that this positive effect of an increased Se level in the milk of cows fed with the SeI ration does not seem to be affected by the high levels of I present in this diet. The level of I analyzed in the milk from the SeI group was also interesting to consider since it was 559% higher at the end of the test than at the initial level. Using a similar approach, Kaufmann and Rambeck [49] determined that a higher I intake increased the I concentration in milk, and the researchers obtained I levels in a range of 400–500 µg/L with an I supplementation of 60–150 mg/day in the feed.

Dietary reference values for Se and I for the adult population may vary; thus, for Se, the Institute of Medicine [90] indicates a population reference intake of 55 µg/day, and the European Food Safety Authority (EFSA) [91] describes an adequate intake of 70 μg/day. For I, the most common population reference intake value in the literature is 150 μg/day for adults [92]. Therefore, 100 mL of milk produced by the SeI group at the end of the study reached 5–6% of the daily reference value for Se, and 80% for I.

According to Regulation 1169/2011 (Articles 32 and 33, and Annex XIII Part A) of the European Union, beverages that provide 7.5% of the daily reference intake levels of Se or I can be labeled as a “source of Se” (≥4.13 µg/100 mL) or “source of I” (≥11.25 µg/100 mL), respectively [93]. In addition, if food contains at least twice the value considered as a “source of”, it can be considered to contain a “high content”. On this basis, we did not achieve an appropriate supplementation level in the milk that could be labeled as a source of Se by the end of the test (the maximum Se level in our experiment was 3.32 expressed as µg/100 mL). These results are in line with the results of previous works [22,89,94], where the achievement of milk Se levels above 4.13 µg/100 mL is successful when cows’ feed is supplemented with more than 0.2 ppm of an organic Se source, and this is not legally permitted in the European Union. As for I, labeled levels were obtained as a source of I in the milk for both groups, both at the beginning and end of the test. These results are reasonable, considering that milk is recognized as a natural source of I in the diet of human beings [95]. In addition, at the end of the study, milk obtained from the control and supplemented groups could be labeled as milk with a “high I content” (≥22.5 µg/100 mL), reaching an I value of 120.2 expressed as μg/100 mL in the SeI group. It is worth noting that this level of I found in 100 mL of milk represents 20% of the maximum daily intake limit in the human diet recommended by the Scientific Committee on Food (SCF) [25] (UL = 600 μg/day). Currently, some authors indicate that levels of I supplementation below the maximum permitted levels would be sufficient to enrich milk in I, without compromising the margin of safety for its consumption [68]. Also, the literature reports highly variable levels of I in milk, influenced by factors including dietary contributions, presence of I antagonists, and management practices, such as the application of iodine-based disinfectants on the udder [68,96]. This variability highlights the importance of informing consumers about the I levels in the dairy products they consume to ensure their proper use in human nutrition.

### 4.4. Dairy Products

Dehydration achieved using the spray-drying method is the most frequently used technique in the field to promote the preservation and stabilization of milk constituents to extend their storage life and later use [97]. Since trace minerals can be lost during heat treatment or other unit operations involving heat, such as evaporation and drying [98], it is important to evaluate the mineral levels in dairy products produced after milk processing. In our study, the spray-dried milk collected from the group supplemented with organic Se and I (SeI ration) could be labeled as a “source of Se”, since, in the European Union, solid food that provides 15% of the daily reference intake of Se (≥8.25 µg/100 g) complies with this rule. Thus, the Se content in the spray-dried milk obtained from the SeI group reached a value of 22.66 µg/100 g, and this dairy product could also be labeled as “high in Se” since it contained more than twice the amount necessary to conform to the source declaration. Furthermore, the I content suitable for the source declaration (≥22.5 µg/100 g of solid food) was evident in the spray-dried milk obtained from both treatment groups, and the samples could also be labeled as “high in I” because they contained ≥45 µg of I/100 g in solid food (245.47 and 1568.65 µg/100 g of spray-dried milk for the control and SeI groups, respectively). Although the mineral values observed in the spray-dried milk are high, we must consider that, at the moment of consumption, the product will be reconstituted or can be used in the manufacture of other products as a food ingredient to create value-added foods [99].

Ultimately, we observed that dietary supplementation with organic Se and I had a positive effect on the levels of both minerals in the fresh cheese, without affecting the chemical composition, pH, cheese yield, water activity, colorimetric parameters, and the levels of the other minerals in the product. This result is in accordance with that of Ianni et al. [80], who observed no changes in the general chemical composition of cheeses following organic Se supplementation at a level of 0.45 mg/kg in total mixed ration, but they obtained a higher Se level in cheeses from the group supplemented with organic Se, achieving 290 µg/kg after 7 days of testing and 306 µg/kg after 120 days. On the other hand, Ling et al. [22] conducted a study in dairy cows in which they evaluated two consecutive feeding periods with different forms of Se inclusion. In the first experimental period, the diet was supplemented with inorganic Se and, after 64 days, the second period of 57 days began in which cows consumed a combination of organic and inorganic Se at levels similar to those in our trial. The cheeses produced in the first experiment reached a Se level of 146 µg/kg, increasing to 361 µg/kg during the second period. Thus, the combination of the two forms of Se in the diet transferred Se to the cheese most effectively. However, in comparison with our results, the cheeses resulting from the dietary treatment in which the two forms of Se were combined, obtained higher levels than those obtained in our trial (361 µg/kg versus 164 µg/kg), although these authors produced a type of semi-mature cheese, unlike our case, which was fresh cheese. In addition, they suggested that the Se transfer coefficient from milk to cheese depended on the type of cheese processing performed since a lower protein content in cheese could be the reason for the lower Se transfer observed in some studies [100,101], supporting the idea that casein micelles could be the most important location for cheese Se, where selenomethionine is non-specifically incorporated [22]. For this reason, Moschini et al. [4] believed that Se was a more suitable element for producing enriched hard cheeses. Despite the fact that a significant fraction of I can be lost during milk processing and cheese manufacturing activities due to its high volatility and solubility properties [17,102,103], fresh cheeses represent a very good source for the enrichment of this element [4] since they retain higher amounts of whey, where most of the I content is found in solution or associated with whey proteins [104]. However, the mineral concentration eventually increases due to moisture loss as maturation progresses, so hard cheeses will always possess higher mineral levels [103].

Taking into account the daily reference intake levels of Se and I for the adult population, 50 g of cheese from the SeI group at the end of the test met the 15% recommended dietary allowance for Se and the 90% recommended dietary allowance for I. Following the European Union regulations, we observed that fresh cheeses produced by both groups could be labeled as sources of Se (≥8.25 µg/100 g of solid food) and I (≥22.50 µg/100 g of solid food). It is important to highlight that the cheese produced by the SeI group presented a Se content of 16.4 µg/100 g, which was very close to the declaration of a high Se content (16.5 µg/100 g of solid food). In addition, a “high I content” (≥45 µg/100 g) was evident in cheeses from both groups, reaching an I value of 269.7 µg/100 g in the supplemented group.

Selenium and I supplementations did not modify the texture profile of the fresh cheeses manufactured in the trial. Only a slight decrease was observed for the adhesiveness parameter in the SeI group. Regarding the sensory profile study, the higher overall acceptability of cheeses obtained from the control group could be related to the milder cow milk flavor assessed in the supplemented group. In contrast, Azorín et al. [59] observed that supplementation with moderate levels of inorganic Se plus organic Se did not affect the cow milk flavor of cheeses, achieving mean overall acceptability values similar to ours.

Experimental diets did not affect the physicochemical properties of the yogurts produced. In contrast, Achanta et al. [105] determined that the Se enrichment of yogurts could reduce the loss of whey, suggestively leading to a better water holding capacity. Similar to the study conducted with the cheese, no differences were observed in Ca, P, Cu, and Zn levels. In addition, yogurts produced by the SeI group exhibited quantitatively higher Se values, but no significant differences were observed. Csapó et al. [106] observed higher levels of Se in their yogurts after feeding cows with Se yeast at a level of 2 mg Se/day. Moreover, significant differences were viewed for the I level, and yogurts produced from both treatments could be labeled as a “source of I” (≥22.50 µg/100 g), and a “high I content” (≥45 µg/100 g) could be specified for yogurts from the SeI group. Taking into account the daily reference intake levels of Se and I for the adult population, 50 g of yogurt from the SeI group at the end of the test would achieve the 58% recommended dietary allowance of I. It should be noted that, with the SeI diet, we obtained I levels much higher than those reported in the literature, although wide variability in the I content of dairy products has been reported [104,107]. Additionally, our level of I supplementation is much higher than that evaluated by other authors [69].

It is important to evaluate the sensory properties of processed yogurts since their enrichment with some minerals can produce certain properties in the final product that make it unsuitable for human consumption. Alzate et al. [108] experienced a metallic odor and observed a pinkish color in Se-enriched fermented milk when the Se concentrations were above 2 µg/g; however, these values were significantly higher than the maximum observed in our experiment. In our tested yogurts, the panelists determined that there were no significant differences in the parameters analyzed between the control and supplemented groups, achieving both a medium and high level of overall acceptance.

## 5. Conclusions

Supplementing the rations of dairy cows with I plus organic and inorganic Se forms at the maximum levels permitted in the European Union showed an increase in both microminerals in milk and cheese; this allows the use of label specifications of “high content in” for fresh milk and yogurt in the case of I and for spray-dried milk for Se and I, obtaining in cheese the Se levels at 99% of the required score but reaching the value in I for this claim. In general, the dietary treatments we evaluated did not alter the physicochemical properties of dairy products or the health and productivity of the cows. When considering the recommended daily intake levels of Se and I for the adult population, in the case of I, a more moderate supplementation level should be studied due to the high transfer activity observed of this element to the milk, and consequently to the cheese and yogurt. These facts have important implications related to the relevance of informing consumers about the levels of Se and I in the milk and dairy products, to ensure their proper use in human nutrition. Future studies would be necessary to evaluate the long-term effects of these dietary supplements on dairy products, the health and welfare of animals, as well as possible carry-over effects to subsequent lactations on a large commercial scale.

## Figures and Tables

**Table 1:** Ration constituents and calculated composition of the test diets.

Components	Content (% DM ^(1)^)
Alfalfa hay	47.62
Barley straw	4.76
Corn grain	19.00
Barley grain	19.00
Soybean meal (47% Crude Protein)	3.97
Dried beet pulp	2.00
Palm oil	1.75
Sodium bicarbonate	0.70
Monocalcium phosphate	0.46
Sodium chloride	0.32
Vitamin-mineral premix ^(2)^	0.30
Urea ^(3)^	0.12
Calculated composition (DM) ^(4)^	
Crude Protein (%)	15.0
Net Energy for Lactation (MJ/kg)	6.5
Neutral Detergent Fiber (%)	38.0

^1^ DM = dry matter. ^2^ Provided (per kg of diet DM): vitamin A, 7992 IU; vitamin D3, 2088 IU; vitamin E, 32 mg; vitamin B12, 0.0029 mg; biotin, 0.0019 mg; niacin, 5.76 mg; zinc, 57.6 mg; iron, 3.64 mg; manganese, 24 mg; cobalt, 0.12 mg; copper, 13.2 mg; iodine, 0.57 mg in control diet (CON) and 5.68 mg of iodine in supplemented diet (SeI); and selenium, 0.57 mg, as sodium selenite in control diet (CON), and 0.34 mg of Se as sodium selenite plus 0.23 mg as organic selenium (*Saccharomyces cerevisiae* CNCM I-3060 (3b8.10), Alltech, Nicholasville, KY, USA) in supplemented diet (SeI). ^3^ Optigen^®^, Alltech, Nicholasville, KY, USA. ^4^ According to FEDNA [29].

**Table 2:** Chemical composition of experimental rations.

Analyzed Composition (% DM ^(1)^)	CON Ration ^(2)^	SeI Ration ^(3)^
Crude Protein	15.1	14.9
Ether Extract	1.62	1.79
Neutral Detergent Fiber	38.1	37.7
Acid Detergent Fiber	24.8	24.6
Lignin Acid Detergent	4.86	4.91
Ash	7.39	7.39
Ca	0.82	0.83
P	0.31	0.33
I (ppm)	0.95	6.58
Se (ppm)	0.56	0.58

^1^ DM = dry matter. ^2^ CON Ration = control ration with inorganic Se and low I level. ^3^ SeI Ration = experimental ration with inorganic and organic Se and high-dose I level.

**Table 3:** Effect of rations on body condition and milk production of lactating cows.

	CON Ration ^(1)^		SeI Ration ^(2)^		SEM ^(3)^	*p*-Value		
Day of Trial	0	64	0	64		R ^(4)^	D ^(5)^	R × D
Body condition ^(6)^	2.80	2.77	3.02	2.85	0.117	0.661	0.357	0.645
Milk yield (L/d)	30.02	29.07	30.63	29.62	1.378	0.835	0.319	0.976

^1^ CON Ration = control ration with inorganic Se and low I level. ^2^ SeI Ration = experimental ration with inorganic and organic Se and high-dose I level. ^3^ SEM = standard error of the mean (*n* = 10 per treatment). ^4^ R = ration. ^5^ D = day. ^6^ Measured in a range of 1–5.

**Table 4:** Effect of rations on mineral and metabolic profile of blood of lactating cows.

	CON Ration ^(1)^		SeI Ration ^(2)^		SEM ^(3)^	*p*-Value		
Day of Trial	0	64	0	64		R ^(4)^	D ^(5)^	R × D
Whole blood								
Ca (mg/100 g)	6.14	7.67	6.37	8.22	0.142	0.194	0.000	0.318
P (mg/100 g)	19.49	19.86	19.59	19.69	0.488	0.972	0.734	0.851
Cu (mg/100 g)	0.031	0.069	0.028	0.068	0.002	0.655	0.000	0.840
Zn (mg/100 g)	0.086	0.211	0.080	0.201	0.009	0.681	0.000	0.846
Se (μg/L)	166.91	195.66	177.07	200.38	5.44	0.508	0.013	0.765
GSH-Px ^(6)^ (U/L)	33,422.85	36,884.57	33,268.57	37,868.57	1775.201	0.909	0.007	0.653
GSH-Px ^(6)^ (U/g Hb)	317.01	328.59	307.35	346.3	9.994	0.843	0.000	0.022
Plasma								
Glucose (mg/dL)	64.48	54.81	64.40	63.48	1.072	0.066	0.038	0.078
TGs ^(7)^ (mg/dL)	44.36	39.13	36.05	57.87	7.991	0.749	0.744	0.596
Urea (mg/dL)	31.75	35.46	31.80	30.68	1.099	0.302	0.504	0.224
Total proteins (g/dL)	7.91	7.54	8.29	7.74	0.184	0.443	0.007	0.538
Cholesterol (mg/dL)	262.83	269.41	275.27	267.24	17.209	0.884	0.965	0.661
TAC ^(8)^ (mmol/L)	0.21	0.23	0.13	0.19	0.044	0.503	0.322	0.684
TOS ^(9)^ (µmol/L)	13.87	16.48	12.87	13.54	0.870	0.277	0.069	0.263
Total T_4_ (µg/dL)	2.78	2.21	2.41	2.52	0.116	0.901	0.177	0.049

^1^ CON Ration = control ration with inorganic Se and low I level. ^2^ SeI Ration = experimental ration with inorganic and organic Se and high-dose I level. ^3^ SEM = standard error of the mean (*n* = 10 per treatment). ^4^ R = ration. ^5^ D = day. ^6^ GSH-Px = glutathione peroxidase. ^7^ TGs = triglycerides. ^8^ TAC = total antioxidant capacity. ^9^ TOS = total oxidant status.

**Table 5:** Effect of ration on somatic cell content (SCC), chemical and mineral composition of milk of cows.

	CON Ration ^(1)^		SeI Ration ^(2)^		SEM ^(3)^	*p*-Value		
Day of Trial	0	64	0	64		R ^(4)^	D ^(5)^	R × D
SCC (×10^3^) cell/ml	60.75	98.83	57.60	116.00	11.424	0.763	0.050	0.662
Ether Extract (%)	3.06	3.54	2.62	4.36	0.195	0.633	0.011	0.126
Crude Protein (%)	2.93	3.36	2.88	3.36	0.042	0.785	0.000	0.726
Lactose (%)	4.86	4.68	4.87	4.68	0.021	0.969	0.000	0.937
Non-fat solids (%)	8.45	8.80	8.39	8.78	0.052	0.652	0.003	0.916
pH	6.67	6.65	6.68	6.64	0.008	0.978	0.076	0.610
Ca (mg/100 g)	102.10	95.32	97.01	99.05	1.305	0.799	0.377	0.109
P (mg/100 g)	70.56	72.26	69.59	80.66	1.009	0.083	0.006	0.033
Cu (μg/100 g)	3.61	1.76	3.33	3.29	0.308	0.327	0.145	0.159
Zn (μg/100 g)	346.72	388.21	365.02	429.40	8.200	0.087	0.005	0.495
Se (μg/100 g)	1.85	1.58	1.95	3.29	0.064	0.000	0.001	0.000
I (μg/100 g)	20.18	25.94	19.69	110.06	5.246	0.001	0.000	0.001

^1^ CON Ration = control ration with inorganic Se and low I level. ^2^ SeI Ration = experimental ration with inorganic and organic Se and high-dose I level. ^3^ SEM = standard error of the mean (*n* = 10 per treatment). ^4^ R = ration. ^5^ D = day.

**Table 6:** Mineral composition of spray-dried milk from cows fed with the experimental treatments.

	CON Ration ^(1)^	SeI Ration ^(2)^	SEM ^(3)^	*p*-Value
Ca (mg/100 g)	1041.52	660.95	149.118	0.190
P (mg/100 g)	703.60	569.02	58.419	0.166
Cu (mg/100 g)	0.20	0.19	0.164	0.534
Zn (mg/100 g)	2.18	1.90	0.622	0.553
Se (μg/100 g)	6.92	22.66	4.769	0.032
I (μg/100 g)	245.47	1568.65	100.418	0.003

^1^ CON Ration = control ration with inorganic Se and low I level. ^2^ SeI Ration = experimental ration with inorganic and organic Se and high-dose I level. ^3^ SEM = standard error of the mean (*n* = 3 per treatment).

**Table 7:** Chemical and mineral composition of fresh cheese according to experimental treatments.

	CON Ration ^(1)^	SeI Ration ^(2)^	SEM ^(3)^	*p*-Value
Dry extract (% FM ^(4)^)	37.3	42.7	1.97	0.061
Crude Protein (% DM ^(5)^)	38.9	35.3	1.78	0.152
Ether extract (% DM)	30.6	36.9	2.82	0.172
Ca (mg/100 g FM)	514.0	505.8	31.98	0.900
P (mg/100 g FM)	314.9	316.7	19.83	0.960
Cu (mg/100 g FM)	0.41	0.50	0.086	0.602
Zn (mg/100 g FM)	2.70	2.47	0.160	0.525
Se (µg/100 g FM)	10.2	16.4	0.76	0.002
I (µg/100 g FM)	74.3	269.7	20.66	0.000
pH	6.87	6.87	0.035	0.964
Yield	16.8	19.3	1.68	0.490
Water Activity Day 0	0.95	0.96	0.003	0.228
Water Activity Day 14	0.96	0.96	0.002	0.943
Colorimetric parameters ^(6)^				
L*	104.1	103.3	0.49	0.483
a*	−2.38	−2.23	0.048	0.210
b*	7.70	8.15	0.237	0.399
Texture profile				
Hardness (N)	22.0	28.8	5.51	0.186
Cohesiveness (dimensionless)	0.79	0.79	0.011	0.987
Adhesiveness (N s)	−0.27	−0.98	0.190	0.078
Elasticity (mm)	0.88	0.88	0.004	0.565
Chewiness (N mm)	15.4	20.5	4.23	0.193

^1^ CON Ration = control ration with inorganic Se and low I level. ^2^ SeI Ration = experimental ration with inorganic and organic Se and high-dose I level. ^3^ SEM = standard error of the mean (*n* = 4 cheeses × 3 batch for each treatment). ^4^ FM = fresh matter. ^5^ DM = dry matter. ^6^ L* = lightness index, a* = red index, b* = yellow index.

**Table 8:** Chemical and mineral composition, syneresis, and CIELAB parameters of yogurt (FM) obtained from cows fed the experimental treatments.

	CON Ration ^(1)^	SeI Ration ^(2)^	SEM ^(3)^	*p*-Value
pH	4.80	4.99	0.235	0.702
Crude Protein (%)	6.82	6.77	0.293	0.942
Ether Extract (%)	2.89	2.58	0.334	0.061
Syneresis (g/100 g)	29.6	22.18	4.58	0.441
Mineral composition				
Ca (mg/100 g)	203.9	192.8	5.27	0.170
P (mg/100 g)	153.1	148.7	4.76	0.324
Cu (mg/100 g)	0.090	0.240	0.054	0.192
Zn (mg/100 g)	0.760	0.738	0.016	0.512
Se (µg/100 g)	3.88	4.53	0.555	0.580
I (µg/100 g)	42.8	176.3	3.18	0.000
Colorimetric parameters ^(4)^				
L*	102.2	102.4	0.823	0.911
a*	−3.22	−3.29	0.283	0.912
b*	9.06	8.84	0.804	0.898

^1^ CON Ration = control ration with inorganic Se and low I level. ^2^ SeI Ration = experimental ration with inorganic and organic Se and high-dose I level. ^3^ SEM = standard error of the mean (*n* = 3 yogurts × 3 batch for each treatment). ^4^ L* = lightness index, a* = red index, b* = yellow index.

## Data Availability

Data are contained in the article.
